# Exome Sequencing and Functional Analysis Identifies a Novel Mutation in *EXT1* Gene That Causes Multiple Osteochondromas

**DOI:** 10.1371/journal.pone.0072316

**Published:** 2013-08-29

**Authors:** Feng Zhang, Jinlong Liang, Xiong Guo, Yingang Zhang, Yan Wen, Qiang Li, Zengtie Zhang, Weijuan Ma, Lanlan Dai, Xuanzhu Liu, Ling Yang, Jun Wang

**Affiliations:** 1 Key Laboratory of Environment and Gene Related Diseases of Ministry Education, Faculty of Public Health, College of Medicine, Xi’an Jiaotong University, Xi’an, Shaanxi, China; 2 BGI-Shenzhen, Shenzhen, China; 3 Department of Orthopedics, First Affiliated Hospital of Medical College of Xi’an Jiaotong University, Xi’an, Shaanxi, China; 4 Department of Orthopedics, First People’s Hospital of Longxi County, Gansu, China; 5 BGI-Tianjin, Tianjin, China; 6 Department of Biology, University of Copenhagen, Copenhagen, Denmark; 7 King Abdulaziz University, Jeddah, Saudi Arabia; University of Texas MD Anderson Cancer Center, United States of America

## Abstract

Multiple osteochondromas (MO) is an inherited skeletal disorder, and the molecular mechanism of MO remains elusive. Exome sequencing has high chromosomal coverage and accuracy, and has recently been successfully used to identify pathogenic gene mutations. In this study, exome sequencing followed by Sanger sequencing validation was first used to screen gene mutations in two representative MO patients from a Chinese family. After filtering the data from the 1000 Genome Project and the dbSNP database (build 132), the detected candidate gene mutations were further validated via Sanger sequencing of four other members of the same MO family and 200 unrelated healthy subjects. Immunohistochemisty and multiple sequence alignment were performed to evaluate the importance of the identified causal mutation. A novel frameshift mutation, c.1457insG at codon 486 of exon 6 of *EXT1* gene, was identified, which truncated the glycosyltransferase domain of *EXT1* gene. Multiple sequence alignment showed that codon 486 of *EXT1* gene was highly conserved across various vertebrates. Immunohistochemisty demonstrated that the chondrocytes with functional EXT1 in MO were less than those in extragenetic solitary chondromas. The novel c.1457insG deleterious mutation of *EXT1* gene reported in this study expands the causal mutation spectrum of MO, and may be helpful for prenatal genetic screening and early diagnosis of MO.

## Introduction

Multiple osteochondromas (MO, OMIM 133700) is an autosomal dominant inherited disease, and is characterized by multiple cartilage-capped benign tumors, short stature and other skeletal disorders that are caused by mechanical compression of adjacent vessels and nerves [1]. MO usually occurs during childhood and gradually increases in sizes and numbers until the end of puberty. The estimated prevalence of MO ranges from 1/100 in a small Guam population to 1/50,000 in Western populations [2], [3]. Some cases of MO will transform into malignant osteosarcoma or chondrosarcoma [3], [4].

Because the molecular mechanism of MO remains elusive, the only effective treatment is surgical resection of continuously emerging chondromas. Identifying causal gene mutations will advance prenatal genetic screening as well as early diagnosis and treatment of MO [5]. MO is genetically heterogeneous and two causal genes have already been identified in previous studies, including *EXT1* located on chromosome 8q24.1 [6], [7] and *EXT2* located on chromosome 11p11 [8], [9]. *EXT1* and *EXT2* belong to the putative tumor-suppressor *EXT* gene family, which also contains three homologous EXT-like genes, including *EXTL1*, *EXTL2* and *EXTL3*. It has been suggested that MO is caused in large part by *EXT1* mutations [10], [11], [12]. The MO caused by *EXT1* gene mutations presents a greater risk for malignant transformation than the MO caused by *EXT2* gene mutations [13], [14]. No linkage evidence has been reported for EXT-like genes.

With the rapid development of DNA sequencing technology, whole exome sequencing is available now. Recent studies have demonstrated the power of exome sequencing for gene mapping of diseases [15], [16], [17], [18]. Exome sequencing should be unbiased and can help to identify novel causal genetic variants, since it does not focus on specific chromosomal regions or genes reported by previous linkage and association studies.

In this study, exome sequencing followed by Sanger sequencing validation, was first used to screen gene mutations in two representative MO patients from a Chinese family. After filtering the data from the 1000 Genome Project and the dbSNP database (build 132), the detected candidate gene mutations were further validated by Sanger sequencing of four other members of the same family and 200 unrelated healthy subjects. Immunohistochemisty and multiple sequence alignment were also conducted to evaluate the importance of the identified causal gene mutation.

## Results

**Figure 1:**
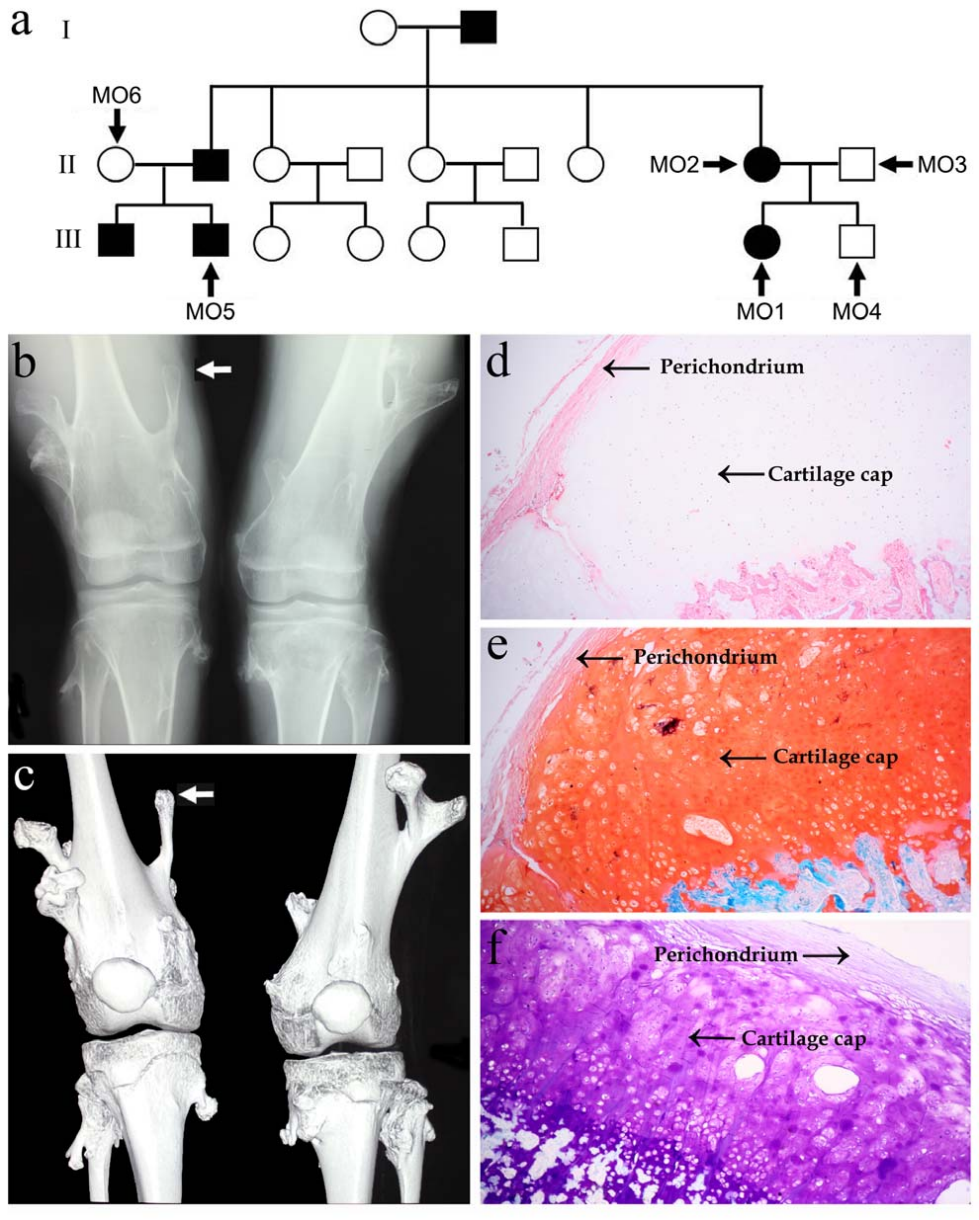
Pedigree structure and characteristic of the MO proband. (a) Pedigree structure of the MO family; (b,c) computed radiography and 3D reconstruction images of knees of the MO proband. The proband exhibits multiple exostoses, arising from the lateral ends of femurs, tibiae and fibulae. Arrowhead denotes the chondroma used for histochemistry staining; (d–f) low-power micrograph (4×) of the proband’s chondroma sections stained by hematoxylin-eosin (d), Safranin O (e) and Toluidine Blue (f). The cartilage cap of MO is covered by fibrous perichondrium and merges into the underlying spongy bone.

The chondroma sections, stained by HE, SO and TB, are presented in Fig. 1d–f. The representative tissue structure of MO was observed with a cartilage cap, covered by fibrous perichondrium, and merged into underlying spongy bone.

On average, exome sequencing generated 7.49 Gb sequence data per sample as 90-bp paired-end reads. After quality control, 92.6% of sequence data was aligned to the UCSC human reference genome (version hg19, build 37.1) and 64.9% of sequence data was mapped to target regions. We achieved an average coverage of 88X and 96% of targeted regions were read more than 10 times, which ensured to detect genetic variants with high sensitivity and specificity. On average, exome sequencing initially identified 104,573 SNPs and 7,335 indels per subject. After removing the common SNPs reported in the dbSNP (build 132) and the 1000 Genome project data, 2,320 SNPs were retained. These variants were further filtered using following criteria: 1) the variants were nonsynonymous and deleterious (as predicted by SIFT software); 2) the variants were shared by all affected family members that underwent exome sequencing. A total of 73 SNPs and 18 indels were retained as candidate causal mutations and were further analyzed.

**Figure 2:**
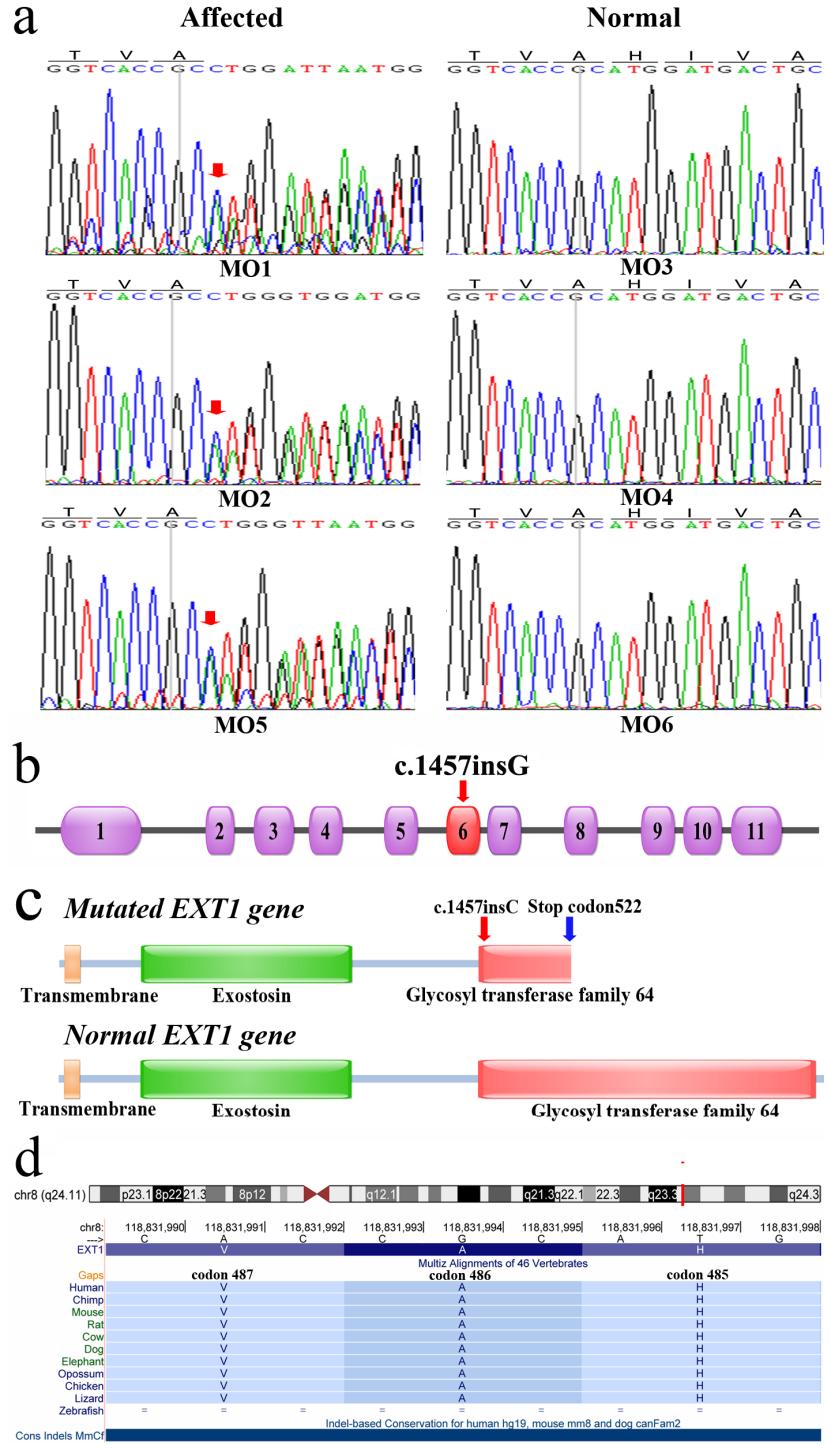
Identification of a frameshift mutation in codon 486 of *EXT1* gene. (a) Sanger sequencing detected the inserted base in the *EXT1* gene of all affected subjects. Red arrowhead denotes the mutation position; (b) intron-exon structure of *EXT1* gene. Mutated exon is indicated by red arrowhead; (c) comparison of the functional domains of EXT1 proteins encoded by mutated and normal *EXT1* genes; (d) multiple sequence alignment of codon 485 to codon 487. Codon 486 is highly conserved across various vertebrates.

The 73 SNPs and 18 indels identified by exome sequencing were further validated by Sanger sequencing of 6 MO family members (including the two MO patients that underwent exome sequencing) and 200 healthy subjects. Only the *EXT1* gene mutation, c.1457insG, was detected in all 3 affected family members, but not in the 3 unaffected family members (Fig. 2a) and the 200 healthy subjects. *EXT1* consists of 11 exons, encoding 746 amino acids (Fig. 2b). The mutation, c.1457insG, occurred at codon 486 of exon 6, causing a frameshift mutation in the glycosyltransferase domain of *EXT1* gene and creating a premature stop codon at amino acid position 520 (Fig. 2c). Codon 486 of *EXT1* gene is highly conserved across various vertebrate species (Fig. 2d), indicating its functional importance.

**Figure 3:**
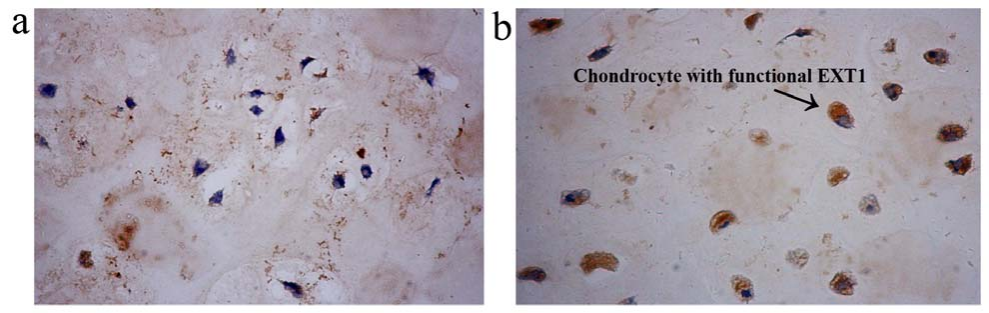
Immunohistochemisty screening of chondrocytes with functional EXT1 in the superficial layers of cartilage caps of MO(a) and extragenetic solitary chondroma(b) (40×). The chondrocytes with functional EXT1 in MO are less than those in extragenetic solitary chondroma.

To assess the functional impact of c.1457insG on EXT1 protein, immunohistochemistry was used to compare EXT1 protein level between a patient with MO and a patient with extragenetic solitary chondromas. In extragenetic solitary chondromas, EXT1 protein was enriched in the cytoplasm of chondrocytes (Fig. 3b). Comparing with extragenetic solitary chondroma, the chondrocytes with functional EXT1 in MO were less than those in extragenetic solitary chondroma (Fig. 3a).

## Discussion

We identified a novel frameshift mutation, c.1457insG in exon 6 of EXT1 gene in this study. The c.1457insG mutation was shared by 3 affected family members, but not by 3 unaffected family members and 200 unrelated healthy subjects. The results of immunohistochemisty and multiple sequence alignment supported the deleterious impact of the c.1457insG mutation on *EXT1* gene. According to the Multiple Osteochondromas Mutation Database (http://medgen.ua.ac.be/LOVDv.2.0/home.php), more than 400 *EXT1* gene mutations have been reported by previous studies. Our results demonstrate the causal role of the c.1457insG mutation and expand the causal mutation spectrum of MO.

The molecular mechanism of MO remains elusive. *EXT1* gene encodes endoplasmic reticulum-resident type II transmembrane glycosyltransferase, which can catalyze the polymerization of heparan sulfate chains at endoplasmic reticulum and Golgi apparatus [19], [20]. Heparan sulfate is an essential regulator of signal transduction during chondrocyte differentiation, ossification and apoptosis [21], [22], such as the diffusion of the Hedgehog protein, and ligand-receptor binding of fibroblast growth factor and bone matrix protein during endochondral ossification [23], [24], [25]. Previous studies suggested that dysfunction of heparan sulfate synthesis would disrupt the Hedgehog, Wingless and Decapentaplegic signaling pathways, and accelerate chondrocyte differentiation [25], [26], [27].

The c.1457insG mutation occurred at the glycosyltransferase domain of *EXT1* gene, which played an important role in the biosynthesis of heparan sulfate [22], [28]. The c.1457insG mutation caused a frameshift at codon 486 and created a premature stop codon at codon 520, which truncated the glycosyltransferase domain of *EXT1* gene. This hypothesis was supported by immunohistochemistry experiment, which observed that the chondrocytes with functional EXT1 in MO were less than those in extragenetic solitary chondroma. This result is consistent with that of a previous study, in which reduced EXT1 protein was observed in MO-derived chondrocytes [29]. One explanation for the reduced EXT1 protein could be nonsense-mediated decay. Even if mutated *EXT1* gene was translated, the products may be unable to correctly fold and would rapidly degrade [29], [30]. Further studies are necessary to clarify whether the c.1457insG mutation leads to nonsense-mediated decay or unstable EXT1 protein.

Based on the results of previous and our studies, we may infer that the MO caused by the c.1457insG mutation was attributed to the functional loss of the glycosyltransferase domain of *EXT1*, which resulted in dysfunction of heparan sulfate biosynthesis [31] and signal transduction. Consequently, chondrocyte proliferation and differentiation are not strictly regulated, leading to the development of MO. Further studies may be necessary to clarify the molecular mechanism of glycosyltransferase domain of *EXT1* gene involved in the development of MO.

In conclusion, we report a novel deleterious frameshift mutation, c.1457insG, in the glycosyltransferase functional domain of *EXT1* gene. The role of the c.1457insG mutation in the development of MO was supported by exome sequencing, Sanger sequencing validation and immunohistochemisty. Our results may be helpful for prenatal genetic screening and early diagnosis of MO.

## Materials and Methods

### Human Subjects

A Han Chinese MO family was investigated in this study (Fig. 1a). The proband (MO1) was a 16-year-old girl suffering from multiple exostoses involving humerus, femur, tibia and fibula (Fig. 1b–c). A group of 200 unrelated healthy subjects, matched for geographical ancestry, were included as controls. Clinical data of each subject was recorded by nurse-administered questionnaires. Each participant underwent careful clinical and computed radiography examination of long bones, truncal and acral joints by two or more experienced orthopedic experts. MO was diagnosed as multiple exostoses, arising from the lateral ends of humerus, ulna, femur, tibiae, fibulae or knee joints. 5 ml EDTA anticoagulated peripheral blood was drawn from 6 family members (Table 1) and from the 200 healthy subjects. Intact chondroma tissues were derived from MO1 and from a patient with extragenetic solitary chondroma (male, aged 3 years) via surgical resection. The collected chondroma tissues were immediately stored in 4% paraformaldehyde solution. This study was approved by the Institutional Review Board (IRB) of Xi’an Jiaotong University. All study subjects or their respective guardians gave their informed written consent by signing a document that had been carefully reviewed by the IRB of Xi’an Jiaotong University.

**Table 1:** Characteristics of study subjects in MO family.

	Age(years)	Sex	MO
MO1	16	Female	Affected
MO2	37	Female	Affected
MO3	38	Male	Unaffected
MO4	17	Male	Affected
MO5	20	Male	Unaffected
MO6	41	Female	Unaffected

### Exome Sequencing

Genomic DNA was extracted from 5 ml peripheral blood, using E.Z.N.A Blood DNA Midi Kit (Omega Bio-tek, Norcross, USA), according to the manufacturer’s protocol. For exome sequencing, 15 µg of extracted DNA was randomly sheared into 250–300 bp fragment libraries by sonication. The purified DNA fragment libraries were captured by NimbleGen 2.1 M capture array (Roche NimbleGen, Madison, USA), followed by 90 bp paired-end sequencing on a Hiseq2000 platform (Illumina, San Diego, USA), according to the manufacturer’s protocol.

### Read Mapping and Variant Analysis

The raw image files of exome sequencing were processed using Illumina Pipeline software (version 1.7) for base calling. The sequences of each subject were generated as 90 bp pair-end reads. SOAPaligner software (http://soap.genomics.org.cn/index.html) was used to align clean reads to the UCSC human reference genome (version hg19, build 37.1, http://genome.ucsc.edu/). Based on SOAPaligner alignment results, SOAPsnp software (http://soap.genomics.org.cn/index.html) was used for SNP calling. BWA software (http://bio-bwa.sourceforge.net/) was used to identify insertions and deletions (indels) in targeted regions by aligning sequencing reads to the UCSC human reference genome. Low-quality SNP calls and indels were filtered out using the following criteria described previously [32]: 1)consensus quality score ≥20; 2) sequencing depth ≥4 and ≤500; 3) copy number ≤2; 4) distance between two adjacent SNPs ≥5 bp. Given that common genetic variations were unlikely to be the causal mutations of MO, all identified SNPs and indels were further filtered against the exome data of 30 Chinese Han individuals from the 1000 Genome Project (ftp://www.1000genome.org) and Chinese Han SNP data available in the dbSNP database (http://www.ncbi.nlm.nih.gov/project/SNP/, build 132). SIFT software (version 4.0, http://sift.jcvi.org/) was used to evaluate the impact of amino acid substitution on protein function and synonymous mutations were removed. Missense mutations and frame-shifting indels that were shared by the two affected family members undergoing exome sequencing, were retained as candidate causal mutations for following study.

### Sanger Sequencing

Sanger sequencing validation was performed in all 6 MO family members (MO1-6) to determine whether the candidate mutations, identified by exome sequencing, co-segregated with MO in the MO family. The population frequencies of the candidate mutations co-segregated with MO were further estimated by Sanger sequencing of 200 unrelated controls with matched geographical ancestry. Sanger sequencing was performed using the standard protocol. Primers used for Sanger sequencing were designed with Primer3 software (http://frodo.wi.mit.edu/).

### Histochemistry and Immunohistochemisty

The paraformaldehyde-fixed chondroma tissues from the MO patient and the extragenetic solitary chondroma patient were rinsed with phosphate buffered saline (PBS), decalcified and embedded in paraffin. Paraffin-embedded chondroma tissues were sectioned (5∼8 µm thick), and placed on glass slides. For histochemistry, the tissue slides of MO were dewaxed in xylene, hydrated with graded ethanol, and stained by hematoxylin-eosin (HE), Safranin O (SO) and Toluidine Blue (TB), respectively.

For immunohistochemisty, the dewaxed and hydrated chondroma sections of MO and extragenetic solitary chondroma, were treated with 3% hydrogen peroxide solution for 10 min, rinsed with PBS, and incubated with rabbit polyclonal anti-EXT1 antibody (1∶50 working dilution, abcam plc, MA, UK) at 4°C overnight. The chondroma sections were then incubated with secondary antibody (ZHONGSHAN golden bridge biotechnology, China) at 37°C for 15 min, exposed to Streptavidin-Horseradish Peroxidase at 37°C for 15 min, and stained with DAB substrate kit (Vector Laboratories, Burlingame, CA) and Meyer hematoxylin.
